# P99 Prevalence of carbapenemase-producing bacteria colonizing hospital surfaces in low- and middle-income countries

**DOI:** 10.1093/jacamr/dlaf230.106

**Published:** 2025-12-04

**Authors:** A Cooke, J Rzaska, R Farzana, B Hassan, C Achi, M Nieto-Rosado, J Oduwo, E Owinoh, R Zahra, K C Iregbu, A Mahboob, S Riaz, M H Hamid, A Muhammad, M Khan, E Rashid, N Medugu, O Oduyebo, I Nwafia, M Yahaya, A Aminu, L Wilson, S Langhorne, A Sindhu, A Rehman, A Shah, A Shoaib, R Tariq, R Temur, M Babar, A Sajid, R Razzaq, S Pervin, R Farzana, N Tasnim, T Akujobi, O Olutekunbi, M Ahmad, P Nwachukwu, I Orabueze, J Abubakar, S Chukwuma, T Walsh, K Sands, K Thomson

**Affiliations:** Ineos Oxford Institute for Antimicrobial Research (IOI), Department of Biology, University of Oxford, Oxford, UK; Ineos Oxford Institute for Antimicrobial Research (IOI), Department of Biology, University of Oxford, Oxford, UK; Ineos Oxford Institute for Antimicrobial Research (IOI), Department of Biology, University of Oxford, Oxford, UK; Department of Medical Microbiology, Cardiff University, Cardiff, UK; Ineos Oxford Institute for Antimicrobial Research (IOI), Department of Biology, University of Oxford, Oxford, UK; Ineos Oxford Institute for Antimicrobial Research (IOI), Department of Biology, University of Oxford, Oxford, UK; Ineos Oxford Institute for Antimicrobial Research (IOI), Department of Biology, University of Oxford, Oxford, UK; Ineos Oxford Institute for Antimicrobial Research (IOI), Department of Biology, University of Oxford, Oxford, UK; Department of Microbiology, Quaid-i-Azam University, Islamabad, Pakistan; National Hospital Abuja, Department of Medical Microbiology, Abuja, Nigeria; Bacha Khan Medical Complex, Swabi, Pakistan; Pakistan Institute of Medical Sciences, Islamabad, Pakistan; Mayo Hospital, Lahore, Pakistan; Lady Reading Hospital, Peshawar, Pakistan; National Institute of Child Health (NICH) and Jinnah Postgraduate Medical Centre (JPMC), Karachi, Pakistan; Department of Microbiology, Quaid-i-Azam University, Islamabad, Pakistan; National Hospital Abuja, Department of Medical Microbiology, Abuja, Nigeria; Massey Street Children Hospital, Lagos, Nigeria; University of Nigeria Teaching Hospital, Enugu, Nigeria; Usmanu Danfodiyo University Teaching Hospital/Sokoto State Specialist Hospital, Sokoto, Nigeria; Aminu Kano Teaching Hospital/Bayero University Kano, Nigeria; School of Energy, Geoscience, Infrastructure and Society, Heriot-Watt University, Edinburgh, UK; Ineos Oxford Institute for Antimicrobial Research (IOI), Department of Biology, University of Oxford, Oxford, UK; Ineos Oxford Institute for Antimicrobial Research (IOI), Department of Biology, University of Oxford, Oxford, UK; Bacha Khan Medical Complex, Swabi, Pakistan; Bacha Khan Medical Complex, Swabi, Pakistan; Pakistan Institute of Medical Sciences, Islamabad, Pakistan; Pakistan Institute of Medical Sciences, Islamabad, Pakistan; Mayo Hospital, Lahore, Pakistan; Mayo Hospital, Lahore, Pakistan; Lady Willingdon Hospital, Lahore, Pakistan; Lady Willingdon Hospital, Lahore, Pakistan; Khulna Medical College Hospital, Khulna, Bangladesh; Dhaka Medical College Hospital, Dhaka, Bangladesh; Mymensingh Medical College Hospital, Mymensingh, Bangladesh; National Hospital Abuja, Department of Medical Microbiology, Abuja, Nigeria; Massey Street Children Hospital, Lagos, Nigeria; Barnards-Balance Laboratory, Murtala Muhammad Specialist Hospital, Kano, Nigeria; University of Nigeria Teaching Hospital, Enugu, Nigeria; Parklane Hospital, Enugu, Nigeria; Usmanu Danfodiyo University Teaching Hospital/Sokoto State Specialist Hospital, Sokoto, Nigeria; Parklane Hospital, Enugu, Nigeria; Ineos Oxford Institute for Antimicrobial Research (IOI), Department of Biology, University of Oxford, Oxford, UK; Ineos Oxford Institute for Antimicrobial Research (IOI), Department of Biology, University of Oxford, Oxford, UK; Ineos Oxford Institute for Antimicrobial Research (IOI), Department of Biology, University of Oxford, Oxford, UK

## Abstract

**Background:**

Antimicrobial resistance (AMR) is one of the fastest growing threats to global human health. Neonates are particularly vulnerable to AMR infections and this problem is exacerbated in LMICs where access to healthcare services and resources including antibiotics are limited. Carbapenem-resistant infections are a major healthcare challenge as carbapenems are often a last-line defence against MDR infections. Bacteria colonizing hospital environments serve as a reservoir of- and potential transmission risk for nosocomial infections.

**Objectives:**

Determine the prevalence of carbapenemase-producing bacterial species colonizing hospital surfaces in neonatal, maternity and delivery wards in 13 hospitals across Bangladesh, Pakistan and Nigeria overall, by surface type and by facility.

**Methods:**

High-touch and low-touch hospital surfaces across 13 hospitals in Bangladesh, Pakistan and Nigeria were sampled on-site using sterile cotton swabs and saline monthly over three consecutive months. Swabs were stored using Amies charcoal media and transported to the UK where they were cultured for 24–48 h at 37°C on antibiotic-supplemented (vancomycin and ertapenem) and non-supplemented chromatic detection agar media (Liofilchem^®^). Species from bacterial growth from non-supplemented agar were identified via MALDI-TOF MS and PCR was employed for samples with growth on agar supplemented with vancomycin and ertapenem to screen for carbapenemase genes *bla*_NDM_, *bla*_KPC_, *bla*_OXA-48-like_, *bla*_VIM_ and *bla*_IMP_.

**Results:**

A total of 1854 hospital surface samples have been processed to date. Microbial growth was obtained from *n*=1636/1854 surface samples. The most common species cultured were *Pseudomonas stutzeri* (*n*=717/1854), *Enterobacter hormaechei* (*n*=165/1854) and *Klebsiella pneumoniae* (*n*=135/1854). Carbapenemase-producing isolates (*n*=744) were found in *n*=476/1854 surface samples, including *bla*_NDM_ (*n*=249/1854), *bla*_KPC_ (*n*=11/1854), *bla*_OXA-48-like_ (*n*=60/1854), *bla*_VIM_ (*n*=221/1854) and *bla*_IMP_ (*n*=82/1854). The surfaces most commonly colonized by carbapenemase producers were in or around sinks including sink bowls/basins, taps and floors surrounding sinks. The species comprising the greatest number of isolates per gene were *K. pneumoniae* for *bla*_NDM_ and *bla*_KPC_ (*n*=59/394 and *n*=7/14, respectively), *K. pneumoniae* for *bla*_OXA-48-like_ (*n*=21/76) and *P. stutzeri* for *bla*_VIM_ and *bla*_IMP_: (*n*=122/263 and *n*=46/93, respectively).

**Conclusions:**

The high contamination rate of hospital surfaces with clinically relevant bacterial species across African and Asian hospitals is concerning. The prevalence of species harbouring carbapenemase genes is also of particular concern in terms of neonatal sepsis as they may be providing a route of transmission for AMR infections to neonatal patients born and treated within the hospitals. Future work will include further hospital surface sampling in addition to performing comparisons between isolates from hospital surfaces and pathogens found to cause neonatal sepsis from the same hospital sites, to investigate potential transmission networks.

